# Metaboloptics: Visualization of the tumor functional landscape via metabolic and vascular imaging

**DOI:** 10.1038/s41598-018-22480-w

**Published:** 2018-03-08

**Authors:** Amy F. Martinez, Samuel S. McCachren, Marianne Lee, Helen A. Murphy, Caigang Zhu, Brian T. Crouch, Hannah L. Martin, Alaattin Erkanli, Narasimhan Rajaram, Kathleen A. Ashcraft, Andrew N. Fontanella, Mark W. Dewhirst, Nirmala Ramanujam

**Affiliations:** 1 Department of Biomedical Engineering, Duke University, Durham, NC USA; 2 Department of Biostatistics and Bioinformatics, Duke University Medical Center, Durham, NC USA; 3 Duke University Medical Center, Durham, NC USA

## Abstract

Many cancers adeptly modulate metabolism to thrive in fluctuating oxygen conditions; however, current tools fail to image metabolic and vascular endpoints at spatial resolutions needed to visualize these adaptations *in vivo*. We demonstrate a high-resolution intravital microscopy technique to quantify glucose uptake, mitochondrial membrane potential (MMP), and SO_2_ to characterize the *in vivo* phentoypes of three distinct murine breast cancer lines. Tetramethyl rhodamine, ethyl ester (TMRE) was thoroughly validated to report on MMP in normal and tumor-bearing mice. Imaging MMP or glucose uptake together with vascular endpoints revealed that metastatic 4T1 tumors maintained increased glucose uptake across all SO_2_ (“Warburg effect”), and also showed increased MMP relative to normal tissue. Non-metastatic 67NR and 4T07 tumor lines both displayed increased MMP, but comparable glucose uptake, relative to normal tissue. The 4T1 peritumoral areas also showed a significant glycolytic shift relative to the tumor regions. During a hypoxic stress test, 4T1 tumors showed significant increases in MMP with corresponding significant drops in SO_2_, indicative of intensified mitochondrial metabolism. Conversely, 4T07 and 67NR tumors shifted toward glycolysis during hypoxia. Our findings underscore the importance of imaging metabolic endpoints within the context of a living microenvironment to gain insight into a tumor’s adaptive behavior.

## Introduction

Early observations of aerobic glycolysis in cancer led to a persistent view that cancers have defective mitochondrial respiration^1^. However, many cancer types have now been shown to rely on mitochondrial metabolism in combination with glycolysis to meet the increased energy demands required for proliferation and metastasis^2–4^. A key indicator of mitochondrial metabolism is the mitochondrial membrane potential (MMP), a transmembrane proton gradient maintained by electron transport^5^, which is frequently increased (i.e. more negative) in a wide range of cancer types^6,7^. Proton pumping during electron transport maintains MMP, which, in the presence of oxygen, can be used to produce adenosine triphosphate (ATP)^8^.

Maintaining increased capacity for both glycolysis and mitochondrial metabolism appears to be critical in helping tumors adapt to environmental stress. In normal tissue, metabolism is directly linked to oxygen availability. Mitochondrial metabolism is preferred during normoxic baseline conditions^9^. During hypoxia, glycolytic proteins are upregulated and metabolic intermediates are shuttled away from the mitochondria in response to hypoxia-inducible factor 1α (HIF-1α) activity^10^. In contrast, some tumors can easily switch between anaerobic and aerobic metabolism without regard for oxygen availability. It is well known that many tumors employ high rates of glycolysis during normoxia (i.e. the Warburg effect)^11,12^. Recent studies also indicate that some aggressive tumor lines heavily utilize mitochondrial metabolism and, unlike primary cells, are able to maintain MMP and mitochondrial metabolism during hypoxia as severe as 0.2-1% O_2_^13,14^. Hypoxia typically causes a HIF-1a mediated increase in mitochondrial autophagy in an attempt to prolong hypoxic survival^10^. Surprisingly, hypoxia has been associated with an increase in mitochondrial mass in metastatic murine breast cancer^15^, and an increase in mitochondrial size mediated by HIF-1α has been shown to prevent mitochondrial apoptosis in colon carcinoma^13^.

It is no surprise that “adaptable” tumors with high capacity for both glycolytic and mitochondrial metabolism under a range of oxygen conditions are better suited to surviving environmental stress, promoting negative outcomes such as increased migration^16^ and metastatic propensity^14^. Recent work also links the adaptable phenotype to metabolic compartmentalization between a tumor and its microenvironment (i.e. the Reverse Warburg Effect)^11^. In the so-called “Reverse Warburg Effect” (RWE), glycolytic stromal cells excrete lactate, and this micro-environmental “waste” is taken in by cancer cells and used to fuel oxidative phosphorylation (OXPHOS)^17^. It follows that observing the regional interplay between multiple metabolic and vascular endpoints aids understanding of a tumor’s phenotype.

Considering the importance of glycolysis, MMP, and the oxygen gradients within blood vessels to tumor bioenergetics, there are surprisingly no techniques to image *in vivo* these three endpoints with a single technology. Commonly used techniques such as cellular metabolic flux analyzers and metabolomics provide comprehensive information about cancer metabolism, but are limited to *in vitro* assays^18^ or *ex vivo* assays^19^ and neither provides spatial information. There are also multiple *in vivo* techniques currently available for metabolic imaging. Positron emission tomography (PET) imaging is widely used to measure glucose uptake with the tracer [^18^F]FDG^20^, and use of additional radio-labeled probes (e.g. [^18^F]FMISO) can also enable detection of tissue hypoxia^21^. The millimeter-scale resolution of PET imaging^21,22^ prevents it from fully capturing tumor heterogeneity at the microscopic level, however. Similarly, magnetic resonance spectral imaging (MR(S)I) can report on a host of important endpoints related to both mitochondrial metabolism and glycolysis^23,24^ as well as vasculature^25^, yet spatial and temporal resolution are limiting^22^.

Vascular imaging can also be accomplished with a range of magnetic resonance imaging (MRI) techniques^20,26^. Blood oxygen level dependent (BOLD) MRI reports on oxygenation^20,27^, dynamic contrast-enhanced (DCE) MRI reports on perfusion and permeability^21,28^, and dynamic susceptibility contrast (DSC) MRI yields angiogenesis and blood volume assessment^29^. However, these must be coupled with additional technologies if metabolic endpoints are desired, and resolution may be too poor for some pre-clinical studies^20,22^.

Optical metabolic imaging has been validated *in vivo* to report on endpoints such as tumor redox status^30–32^ and specifically labeled metabolites^6,33,34^. Optical imaging also allows label-free vascular imaging via endogeneous contrast from hemoglobin^35–37^. Further, of all the available metabolic imaging techniques, only optical imaging provides the micron-scale resolution necessary to visualize both the heterogeneous metabolic landscape and the aberrant vasculature within small tumors^22^. Importantly, *in vivo* optical imaging has not yet been leveraged to perform a comprehensive study measuring glycolysis, mitochondrial metabolism, and small vessel oxygen gradients that influence the overall bioenergetics of a tumor.

We now demonstrate a non-destructive, multi-parametric, intra-vital microscopy technique to image important features of tumor metabolism and vascular physiology at high resolution in small animal models. Glucose uptake is imaged using the well-established indicator 2-[N-(7-nitrobenz-2-oxa-1, 3-diaxol-4-yl) amino]-2-deoxyglucose (2-NBDG) using protocols previously described by our group^38,39^ and others^40–42^. Mitochondrial membrane potential (MMP) is imaged with tetramethylrhodamine, ethyl ester (TMRE)^43–46^. In this manuscript, we thoroughly validate TMRE in normal tissue and tumors to present an optimized method for *in vivo* mitochondrial membrane potential imaging. Oxygen saturation (SO_2_) and vessel architecture are quantified by imaging the differential absorption spectra of oxygenated and de-oxygenated hemoglobin, which is an extensively validated method^47–52^. The relationships between MMP and SO_2_ and glucose uptake and SO_2_ were leveraged in our study to characterize the *in vivo* phenotypes of three murine breast cancer lines- metastatic 4T1 and non-metastatic 67NR and 4T07- that arose from the same parental tumor^53,54^. Using these well-studied sibling tumor lines with unique metastatic properties allowed us to confirm concordance with previous findings and to add new insights using the distinct capabilities of our technology. This work, incorporating two combinations of two endpoints, represents a critical step toward fully integrated three-endpoint metabolic imaging.

Consistent with previous work, optical microscopy demonstrated that the 4T1 tumors displayed a classic Warburg effect, with increased glucose uptake at all SO_2_ levels. However, imaging TMRE with our method also enabled the novel finding that 4T1 have hyperpolarized mitochondria relative to normal tissue, suggesting that both mitochondrial metabolism and glycolysis are increased in 4T1 during baseline conditions. On the other hand, 4T07 and 67NR tumors displayed increased mitochondrial membrane potential relative to normal tissue, but comparable levels of glucose uptake, typical of an oxidative phenotype. The increased MMP in all tumor groups was most pronounced at lower oxygen levels, suggesting that low SO_2_ regions in tumors were associated with increased oxygen-consuming metabolism.

Previous studies have shown that angiogenic vasculature can extend far beyond the tumor border^55^, recruiting fibroblasts^56^ and in turn affecting regional metabolism. We saw that the 4T1 peritumoral areas (PAs) had a distinct hypermetabolic phenotype relative to the tumors themselves; this was not observed for 67NR or 4T07 PAs. Specifically, the 4T1 PAs were characterized by angiogenic vasculature, increased glucose uptake, and decreased MMP relative to the neighboring tumors. We hypothesized that increased regional metabolic cooperation would allow 4T1 alone to maintain robust MMP during hypoxia. Indeed, under hypoxic stress, 4T1 tumors showed a significant increase in MMP, with a corresponding significant drop in the SO_2_, indicative of intensified mitochondrial metabolism. Conversely, 4T07 and 67NR tumors shifted toward a glycolytic phenotype. These findings highlight the unique capability of *in vivo* metabolic and vascular imaging to provide insight into the microenvironment’s influence on tumor metabolic phenotype.

## Results

### TMRE responds to established perturbations of mitochondrial membrane potential *in vivo*

**Figure 1:**
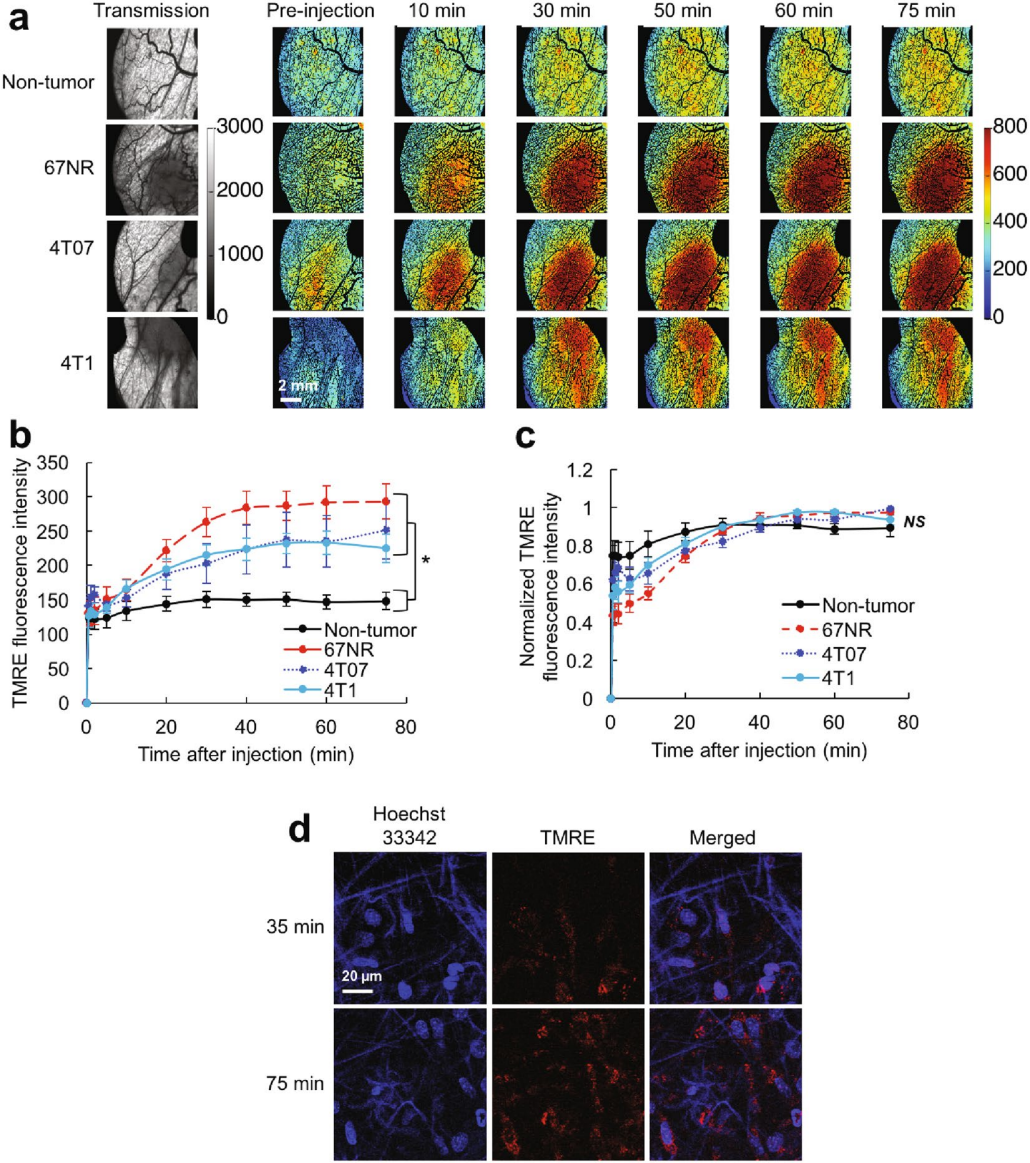
TMRE *in vivo* uptake kinetics are robust across normal and tumor groups. (**a**) Representative images of TMRE fluorescence in non-tumor and in 67NR, 4T07, and 4T1 tumor window chambers. Baseline images were acquired prior to TMRE injection. Tumor regions are shown in the transmission images as areas of increased absorption contrast. **(b**) Mean TMRE uptake kinetics for the non-tumor and tumor groups. (**c**) Kinetics for a given mouse were normalized to the mouse’s max TMRE fluorescence during the imaging period, and normalized kinetics were then averaged within a group. (**d**) Multiphoton imaging of TMRE and Hoechst 33342 in a non-tumor window. Images show two fields of view in the same animal. n = 6 mice (all groups). N.T. = Non-tumor. Error bars = SE. *for p < 0.05.

**Figure 2:**
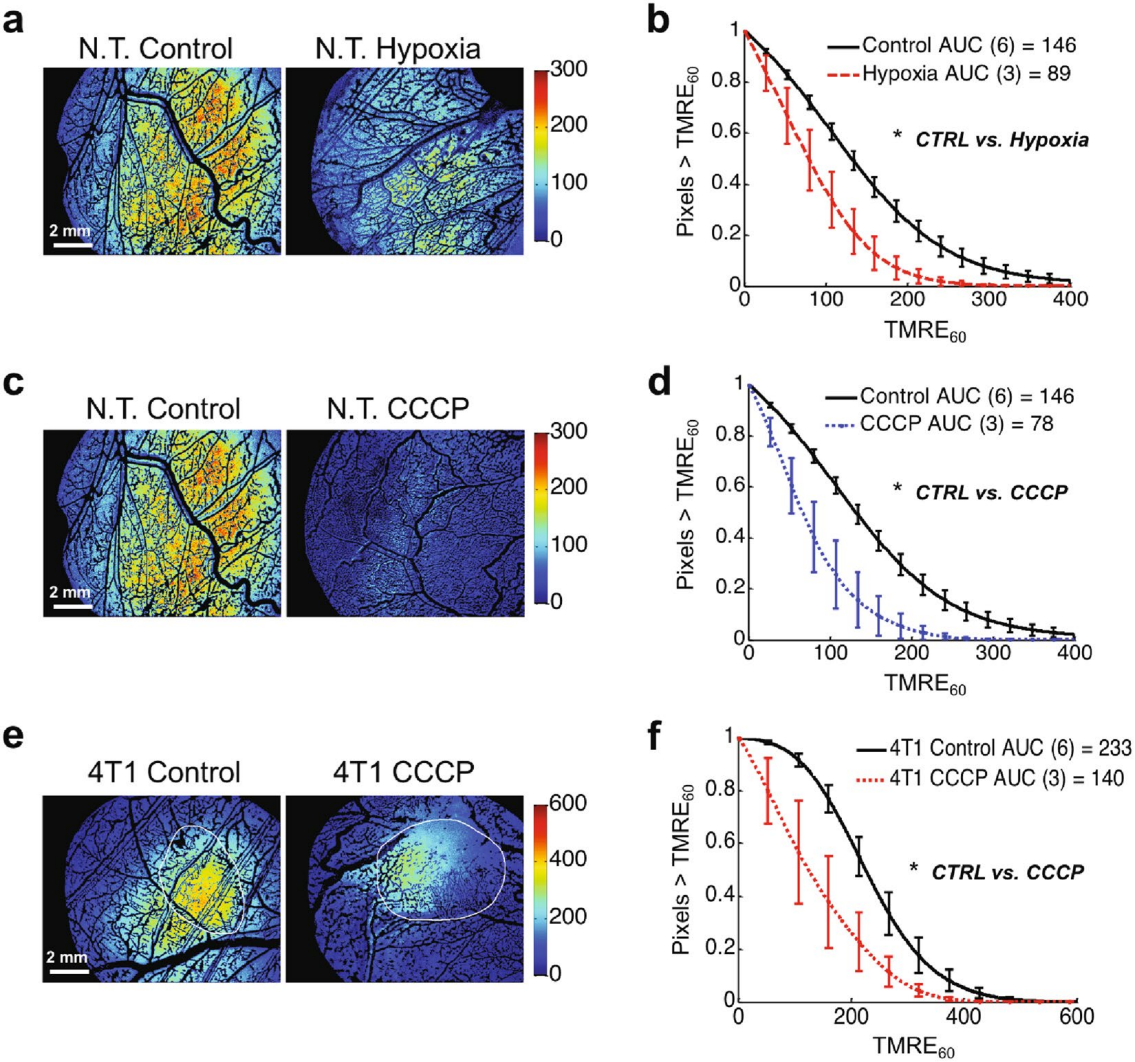
TMRE responds to established perturbations of mitochondrial membrane potential *in vivo* in non-tumor and 4T1 tumor windows. Representative images (**a**) and mean distributions (**b**) of all TMRE uptake at 60 minutes (TMRE_60_) pixels for non-tumor mice with control TMRE imaging or TMRE imaging during forced hypoxia (10% inspired O_2_). Representative images (**c**) and mean distributions (**d**) of all TMRE_60_ pixels for non-tumor mice with control TMRE imaging or TMRE imaging after CCCP pre-treatment. Representative images (**e**) and mean distributions (**f**) of all TMRE_60_ pixels for 4T1 tumor-bearing mice with control TMRE imaging or TMRE imaging after CCCP pre-treatment. Group numbers shown in legend. N.T. = Non-tumor. AUC = area under curve. Error bars = SE. *is p < 0.05.

Figures 1 and 2 demonstrate a systematic investigation of TMRE uptake properties and the effects of perturbing mitochondrial membrane potential on TMRE uptake. Fig. 1a shows TMRE imaging in both tumor and non-tumor (N.T.) window chambers, which is quantified into TMRE uptake curves in Fig. 1b. TMRE uptake kinetics were significantly different in all tumor types compared to N.T. windows, due to increased TMRE uptake in the tumor groups (p < 0.05 for 67NR, 4T07, or 4T1 vs. normal). No difference was seen between tumor groups. Normalizing each uptake curve to its respective TMRE peak demonstrates that both tumor and N.T. groups reach a similar uptake plateau by 40 minutes post-injection (Fig. 1c). TMRE uptake is stable from t = 40–75 minutes post-injection in both normal tissue and tumors, and can thus be measured at any point during the stable imaging window. Fig. 1d shows multi-photon imaging of TMRE and Hoechst 33342 (nuclear stain) in a N.T. window chamber at two time points representing the start and end of the stable window. By 35 minutes post-injection, TMRE signal was localized to mitochondrial-sized features surrounding cell nuclei, and this staining pattern persisted until the end of imaging (75 minutes post-injection). Red-channel background fluorescence was negligible at both timepoints, and non-nuclear signal in the blue channel resulted from second harmonic generation of collagen. The known high binding affinity of TMRE [1] and the multiphoton imaging of TMRE’s mitochondrial localization together indicate that TMRE signal results primarily from mitochondrial regions.

Three distinct mitochondrial perturbations were tested in non-tumor window chambers. Hypoxia is known to elicit a decrease in mitochondrial metabolism^10^. Fig. 2a suggests a decrease in TMRE uptake in N.T. windows during hypoxic (10% inspired O_2_) compared to control (21% inspired O_2_) conditions. Fig. 2b shows that the distribution of all TMRE_60_ pixels for the hypoxia group was significantly decreased relative to the control distribution (p < 0.05), as expected. Another group of N.T. window chambers was treated with CCCP, which is known to dissipate mitochondrial membrane potential^6^, and representative images in Fig. 2c show a qualitative decrease in TMRE uptake in response to CCCP-treatment. Fig. 2d demonstrates that the distribution of all TMRE_60_ pixels for the CCCP group was significantly decreased relative to control (p < 0.05). Similarly, when 4T1 tumor-bearing window chambers were treated with CCCP, TMRE uptake decreased significantly relative to control (p < 0.05), as shown in the images in Fig. 2e and pixel distribution curves in Fig. 2f. The injection volume and concentration of TMRE were kept consistent across all experiments.

### Metabolic and vascular imaging of murine sibling tumor lines of breast cancer

**Figure 3:**
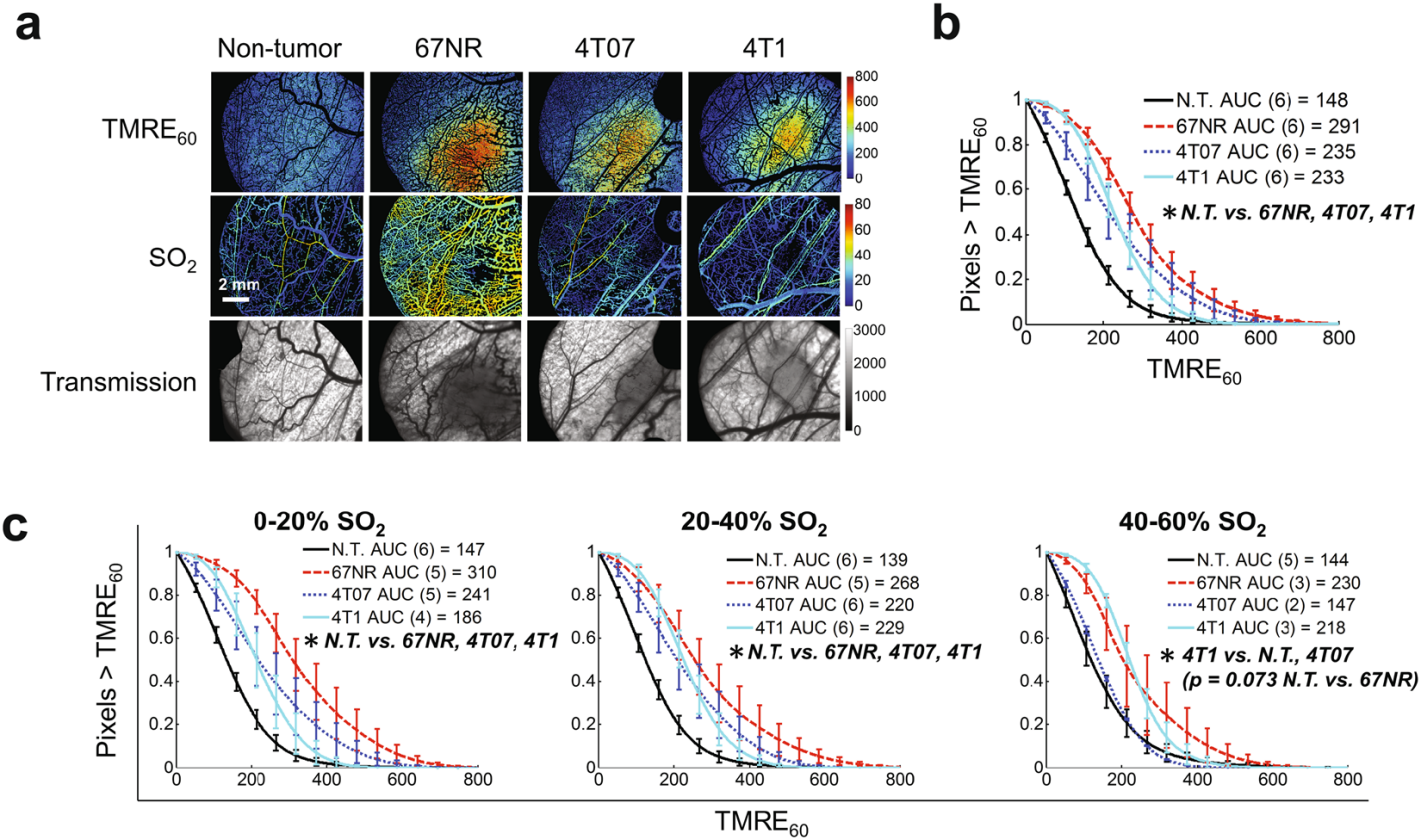
All tumor types show comparable mitochondrial membrane potential (MMP), and low vascular oxygenation (SO_2_) enhances differences between tumor and normal MMP. (**a**) TMRE uptake at 60 minutes (TMRE_60_) and SO_2_ in non-tumor and tumor window chambers. Tumor regions are shown in the transmission images as areas of increased absorption contrast. (**b**) Mean distribution of all TMRE_60_ pixels for each tissue type. (**c**) Mean distribution of TMRE_60_ for each tissue type for distinct levels of regional SO_2_. Group numbers shown in legend. N.T. = Non-tumor. AUC = area under curve. Error bars = SE. *is p < 0.05.

Figure 3 explores the effects of regional SO_2_ on the distribution of TMRE_60_ in tumor and N.T. windows. Typical images of TMRE_60_ and SO_2_ in Fig. 3a highlight increased TMRE uptake in all tumor types compared to N.T. windows. Transmission images show increased absorption in tumor regions. Fig. 3b shows the distribution of all TMRE_60_ pixels for each tissue type. TMRE_60_ was increased in all tumor types relative to N.T. (p < 0.05 N.T. vs. 67NR, 4T07, or 4T1). Fig. 3c takes advantage of the spatial relationships obtained by imaging to show the distribution of TMRE_60_ at distinct levels of SO_2_. TMRE_60_ was increased in tumor relative to N.T. at 0–20% SO_2_ (all tumor v. N.T. p < 0.05) and 20–40% SO_2_ (all tumor v. N.T. p < 0.05). At 40–60% SO_2_, TMRE_60_ was greater in 4T1 than 4T07 or N.T. (p < 0.05), and greater in 67NR than N.T. (p = 0.073). N.T. and 4T07 were indistinguishable for 40–60% SO_2_. Oxygen consumption rates were comparable in 67NR and 4T1 cells when measured by a Seahorse mitochondrial assay (Supplementary Fig. S2).

**Figure 4:**
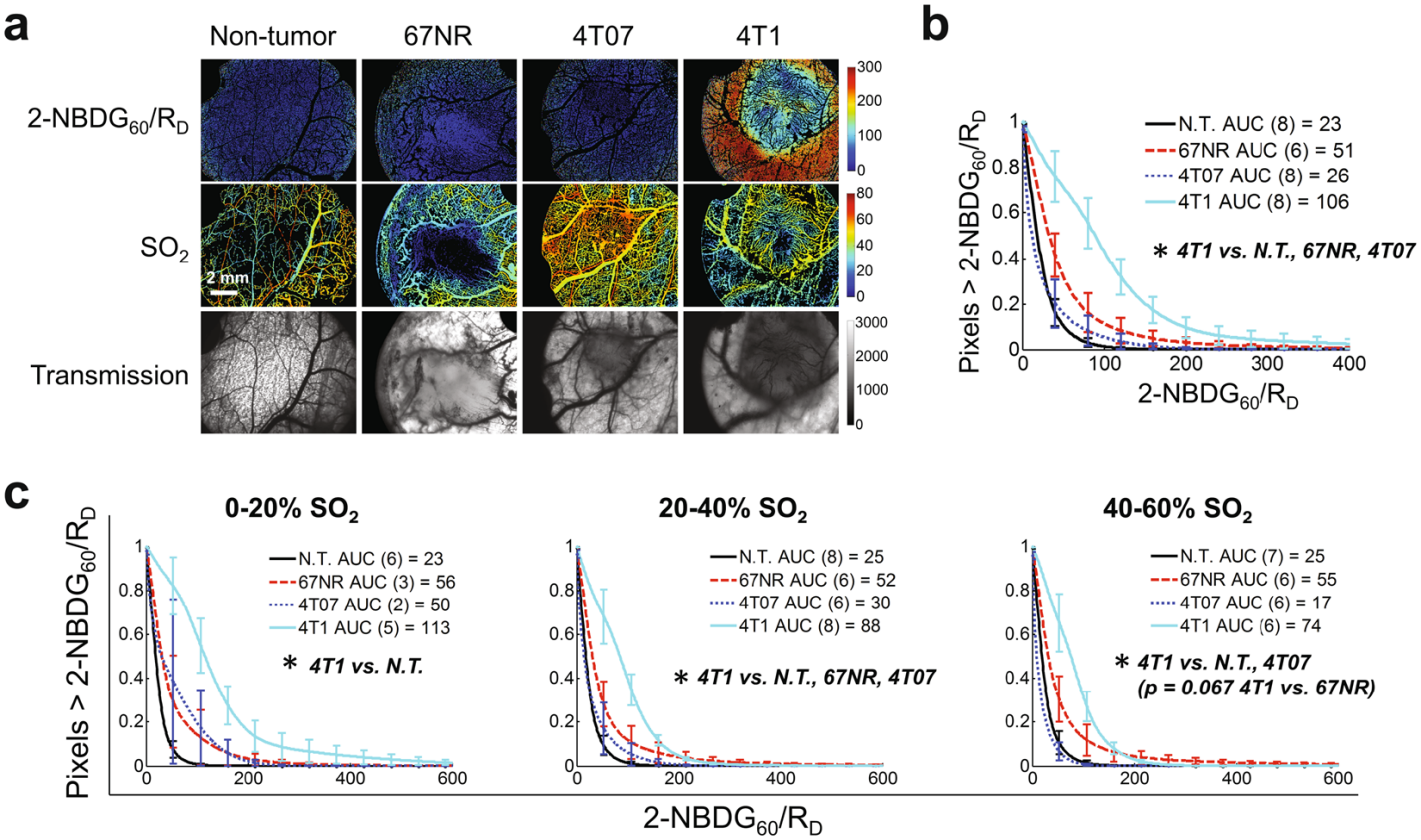
Glucose uptake is increased in 4T1 regardless of regional oxygenation. (**a**) Glucose uptake (2-NBDG_60_/R_D_) and vascular oxygenation (SO_2_) in a non-tumor window chamber and non-metastatic (67NR and 4T07) and metastatic (4T1) tumor window chambers. Tumor regions are shown in the transmission images as areas of increased absorption contrast. (**b**) Mean distribution of all 2-NBDG_60_/R_D_ pixels for each tissue type. (**c**) Mean distribution of 2-NBDG_60_/R_D_ for each tissue type for distinct levels of regional SO_2_. Group numbers shown in legend. N.T. = Non-tumor. AUC = area under curve. Error bars = SE. *is p < 0.05.

Glucose uptake (2-NBDG_60_/R_D_^38^) was imaged in tumor and N.T. window chambers (Fig. 4a). Fig. 4b shows that the distribution of all 2-NBDG_60_/R_D_ pixels for 4T1 was dramatically increased relative to all other groups (p < 0.05 4T1 vs. N.T., 67NR, or 4T07). All other comparisons were not significant. Fig. 4c shows that the increased 2-NBDG_60_/R_D_ in the 4T1 tumors was maintained regardless of SO_2_ level. 2-NBDG_60_/R_D_ was increased in 4T1 relative to N.T. at all SO_2_ (p < 0.05). 2-NBDG_60_/R_D_ was increased in 4T1 relative to both other tumor lines at 20–40% SO_2_ (p < 0.05 4T1 v. 67NR or 4T07) and at 40–60% SO_2_ (4T1 v. 67NR p = 0.067, 4T1 v. 4T07 p < 0.05), but not at 0-20% SO_2_. Surprisingly, N.T., 67NR, and 4T07 were indistinguishable at all SO_2_ levels. Results were consistent with a Seahorse assay that showed increased lactate production in 4T1 (Supplementary Fig. S2).

### Characterization of the metabolic and vascular landscape in the peritumoral area

**Figure 5:**
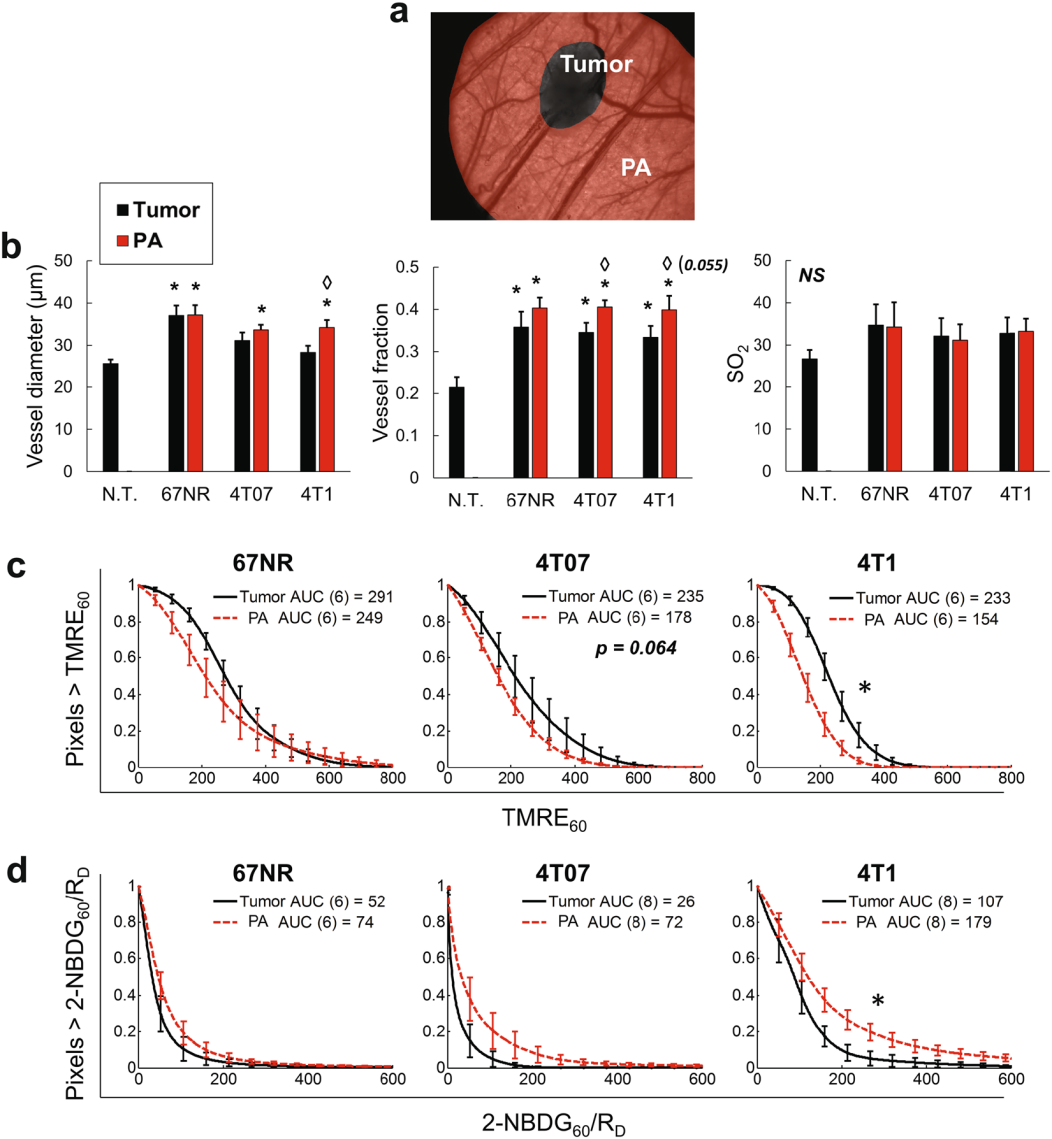
4T1 tumor and peri-tumoral area maintain distinct metabolic and vascular phenotypes. Metabolic and vascular endpoints were quantified for the tumor and peritumoral area (PA) of all tumor types (67NR, 4T07, 4T1). (**a**) Transmission images were segmented into tumor and peritumoral area (PA) regions. (**b**) Comparison of mean vascular features in non-tumor tissue and tumor and PA regions of all tumor types. SO_2_ = vascular oxygenation. (**c**) TMRE_60_ in the tumor and PA of all tumor types. (**d**) 2-NBDG_60_/R_D_ in the tumor and PA of all tumor types. N.T. = Non-tumor. AUC = area under curve. Error bars = SE. n = 12-14 (**b**) or as shown in legend (**c**,**d**). *is p < 0.05 vs. N.T. (**b**). ◊ is p < 0.05 tumor vs. PA for same tumor line (**b**). *is p < 0.05 tumor vs. PA for same tumor line (**c**,**d**).

Previous studies have shown that vasculature can be significantly altered in tissue extending well beyond the tumor border^55^. We segregated each tumor window image into two regions - the tumor and the peritumoral area (PA) - to examine the respective vascular features. As shown in Fig. 5a, transmission images were used to hand-mask tumor regions, and PA was automatically identified as all tissue <5 mm from the tumor^57^ and >1 mm from the image edge, to avoid artifact. In Fig. 5b, vessel diameter and vessel fraction (the percentage of vascular pixels, i.e. # vascular pixels/# total pixels) in both tumor and PA were consistently differentiated from N.T. Mean SO_2_ was comparable across groups. The PA of 4T1 and 4T07 (but not 67NR) showed significantly higher vessel fraction compared to the tumor itself (p < 0.05 for 4T07 and p = 0.055 for 4T1). The 4T1 PA also showed a significant increase in vessel diameter relative to the corresponding tumor region (p < 0.05).

The atypical vascular phenotype of the PAs led us to compare both TMRE_60_ and 2-NBDG_60_/R_D_ in tumor and PA regions. In Fig. 5c,d, TMRE_60_ was significantly decreased and 2-NBDG_60_/R_D_ was significantly increased in the 4T1 PA relative to the 4T1 tumor (both p < 0.05). 2-NBDG_60_/R_D_ also showed a borderline increase in the 4T07 PA relative to the 4T07 tumor (p = 0.07). No differences were seen in 67NR tumor and 67NR PA. The PAs were additionally parsed into radial 1 mm regions to determine the effects of distance on vascular and metabolic properties in the PAs, as shown in Supplementary Fig. S3.

### The Effect of Forced Hypoxia on Tumor Metabolism

**Figure 6:**
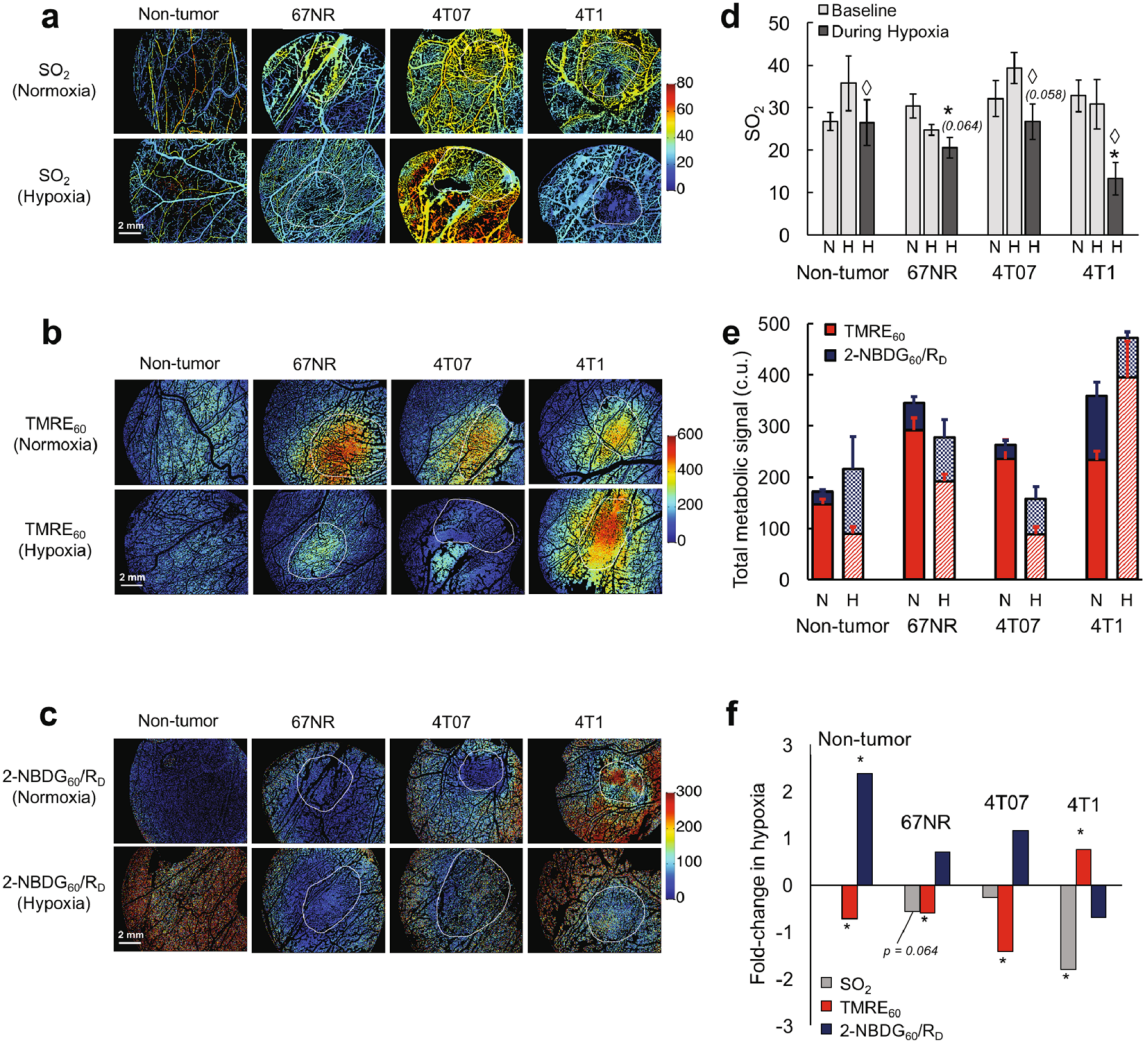
Forced hypoxia reveals a switch toward mitochondrial metabolism in 4T1 tumors and toward glycolysis in 67NR and 4T07 tumors and non-tumor tissue. Images of SO_2_ (%) (**a**) TMRE_60_ (**b**) and 2-NBDG_60_/R_D_ (**c**) during normoxic (21% O_2_) and hypoxic (10% O_2_) conditions. (**d**) Mean SO_2_ for normoxia (N) and hypoxia (H) animal groups. SO_2_ was measured at baseline (light gray, measured in N and H groups) and during hypoxia (dark gray, measured in H group only). (**e**) Total metabolic signal (2-NBDG_60_/R_D_ + TMRE_60_ fluorescence intensities in calibrated units (c.u.)) during normoxia (N) and hypoxia (H). Red and blue show contributions of TMRE and 2-NBDG, respectively, to the total metabolic signal. (**f**), Fold-change in mean TMRE_60_, 2-NBDG_60_/R_D_, and SO_2_ during hypoxia compared to normoxia (fold-change = Mean_HYPOXIA_/Mean_NORMOXIA_). Normoxia: n = 6–8 (TMRE_60_ and 2-NBDG_60_/R_D_) and n = 11–14 (SO_2_). Hypoxia: n = 3 (TMRE_60_ and 2-NBDG_60_/R_D_) and n = 5–6 (SO_2_). N.T. = Non-tumor. Error bars = SE. Tumor outlines shown in white. *is p < 0.05 vs. normoxsia group (D,F). ◊is p < 0.05 vs. hypoxia group baseline (**d**).

We next asked whether the metabolic compartmentalization seen in 4T1 tumors and their PAs would correlate with improved ability to maintain mitochondrial metabolism during hypoxia (i.e. Reverse Warburg Effect). Representative images of metabolic endpoints during normoxic baseline conditions (21% inspired O_2_) and during forced hypoxia (10% inspired O_2_) are shown in Fig. 6a (SO_2_), Fig. 6b (TMRE_60_), and Fig. 6c (2-NBDG_60_/R_D_). Note that the changes observed in 4T1 during hypoxia are qualitatively distinct from those seen in all other groups. Mean SO_2_ for the normoxia and hypoxia groups is shown in Fig. 6d. SO_2_ was quantified at baseline in mice belonging to both the normoxia and hypoxia groups (shown in light gray). SO_2_ was also measured during hypoxia, 15 minutes after the induction of hypoxic breathing, in the hypoxia group (shown in dark gray). SO_2_ decreased in 4T1 (p < 0.05), 4T07 (p = 0.058), and N.T. (p < 0.05) during hypoxia compared to the animal-matched baseline measurements. SO_2_ decreased in 4T1 (p < 0.05), and to a lesser extent in other groups (p = 0.064 for 67NR), when comparing the hypoxia group (during hypoxia) to the normoxia group. Baseline SO_2_ was statistically indistinguishable between the normoxia and hypoxia groups for N.T. (p = 0.12), 67NR (p = 0.20), 4T07 (p = 0.33), and 4T1 (p = 0.79).

The total metabolic signal for each group during normoxia and hypoxia is quantified in Fig. 6e, where total metabolic signal is the sum of calibrated 2-NBDG_60_/R_D_ and TMRE_60_ fluorescence intensities. Red and blue portions of the bar show contributions of TMRE and 2-NBDG, respectively, to the total metabolic signal, to allow for qualitative visualization of metabolic changes during hypoxia. It is evident that there is a shift towards increased glycolysis in N.T., 4T07 and 67NR tumors while there is an increase in mitochondrial metabolism in the 4T1 tumors under hypoxic stress.

For each endpoint in Fig. 6d and e, the fold-change from normoxia to hypoxia was calculated as fold-change = Mean_HYPOXIA_/Mean_NORMOXIA_ and shown in Fig. 6f. Significance was established with a two-sided t-test that compared the normoxic and hypoxic groups for each endpoint. N.T., 67NR, and 4T07 all experienced a decrease in TMRE_60_ (all p < 0.05), and an increase in 2-NBDG_60_/R_D_ (p < 0.05 for N.T.). 67NR also experienced a borderline decrease in SO_2_ (p = 0.064). On the other hand, 4T1 experienced a significant increase in TMRE_60_ and decreases in SO_2_ (p < 0.05) and 2-NBDG_60_/R_D_ (p = NS). Changes in TMRE_60_ and 2-NBDG_60_/R_D_ were complementary; when one endpoint decreased during hypoxia, the other increased without exception. The full pixel distributions for endpoints (SO_2_, TMRE_60_, and 2-NBDG_60_/R_D_) are shown for all normoxia and hypoxia groups in Supplementary Fig. S4.

## Discussion

Considering the popularity of cancer metabolism for diagnosis, prognosis, and treatment, there is a surprising lack of *in vivo* metabolic imaging strategies that can be easily incorporated into pre-clinical studies. Current gold standards for *in vivo* vascular and metabolic imaging, though improving, lack the spatial and temporal resolution needed to examine local tumor heterogeneity^22^. Metabolomics^19^ and Seahorse extracellular flux analyzer^18^ assays provide invaluable information, but are inherently destructive and therefore not amenable to examining the tumor landscape. There is a need for complementary tools that can bridge the gap between multi-parametric *ex vivo* analysis and low-resolution whole body imaging; *in vivo* reporters of mitochondrial activity are particularly needed. To address these gaps, we designed a metabolic imaging strategy that incorporates endpoints for glucose uptake (2-NBDG_60_/R_D_^38^), mitochondrial membrane potential (TMRE_60_), and vascular oxygenation (SO_2_^38,39^), all imaged using the same technology and in two unique combinations (2-NBDG + SO_2_, TMRE + SO_2_).

We first validated TMRE for use *in vivo*. TMRE reports MMP by accumulating as the charge across the mitochondrion becomes more negative, thus causing an increase in fluorescence intensity^58^. TMRE was chosen for the study because it has several benefits such as increased mitochondrial binding and rapid equilibration^59^, which makes it particularly suitable for *in vivo* studies. The few published studies that use TMRE *in vivo*^46,60^ beautifully demonstrate the probe’s utility, but the studies are typically qualitative and focused on other endpoints. Our study expands upon this work by including a recommended TMRE dose, providing TMRE uptake kinetics in both normal and tumor models, and showing that TMRE responds to multiple validated perturbations. Multiphoton imaging of TMRE’s mitochondrial localization indicated that TMRE signal resulted primarily from mitochondrial regions, and administration of hypoxia and CCCP in normal and/or tumor window chambers diminished TMRE uptake, consistent with expectations from literature^6,10^. Our group also recently demonstrated that TMRE uptake decreased in N.T. window chambers after treatment with 2-deoxyglucose (2-DG)^61^. 2-DG is a well-known inhibitor of glucose utilization^62^ which has been shown to cause loss of mitochondrial membrane potential^63^. In the same study, no change in MMP was seen after administration of glucose^61^.

Increased MMP in tumor relative to normal tissue was consistent with previous literature that indicates hyperpolarized mitochondria are common across many cancers^6,7,64^. Regional analysis showed that the trend was driven by tumor regions with low (0–20%) and intermediate (20–40%) SO_2_ levels, at which tumor and normal tissues were most easily distinguished. Our previous work with window chamber and flank tumor models indicates that an average SO_2_ of 20–40% is expected in these lines^38,65,66^. Analysis confirmed that the increased TMRE uptake in tumors relative to non-tumor tissue was not a function of altered vascular properties in the tumor windows. Pearson’s correlation tests revealed that mean TMRE_60_ was not correlated with vessel fraction (R^2^ = 0.017, p = 0.55), nor was TMRE_60_ correlated with vessel diameter (R^2^ = 0.028, p = 0.43; data not shown). Further, initial uptake kinetics and fluorescence plateau times were observed to be similar between normal and tumor groups, indicating that probe delivery was not impaired in either the tumor or non-tumor group.

Based on previous studies relating increased MMP with aggressiveness^3,67–69^, we hypothesized that MMP would correlate with metastatic potential in our study (4T1 > 4T07 > 67NR), but MMP was comparable across tumor types. The comparable MMP observed here contrasts with previous studies in the same cell lines that showed that TCA cycle intermediates increased with metastatic potential^70^ and that 67NR cells had decreased oxygen consumption relative to 4T1 cells^2^. Our studies did not indicate distinct differences in overall OXPHOS, since MMP and SO_2_ were consistent across tumor groups and oxygen consumption rates were equally robust in 67NR and 4T1 cells when measured by a Seahorse mitochondrial assay. It is notable that our study measures MMP *in vivo*, compared with the TCA cycle intermediate study, which was performed *in vitro*^6^. Further investigation would be needed to pinpoint the difference between the studies, however, previous work has shown stark differences in the *in vitro* and *in vivo* metabolic phenotypes of cell lines including MDA-MB-231 and SiHa^71^. Further, the Simoes Seahorse study utilized cell media with increased glucose and glutamine concentrations (25 mM glucose and 6 mM glutamine^2^) compared to our Seahorse study (12.5 mM glucose and 2 mM glutamine), which could have unknown effects on the observed phenotypes.

A validated surrogate of glucose uptake, 2-NBDG, enabled us to test the hypothesis that 4T1 tumors could utilize glycolysis more readily than their non-metastatic counterparts, as previously seen during hypoxia *in vitro*^2^. The 4T1 displayed a classic Warburg effect, with increased glucose uptake at all SO_2_ levels while glucose uptake was consistently low in N.T., 67NR, and 4T07 tumors. Importantly, mean glucose uptake was not sufficient to distinguish between 4T1 and 67NR, but comparing the distributions of glucose uptake did allow for differentiation between these two groups, highlighting the significance of obtaining spatial data. The increased glucose uptake in 4T1 was maintained across all ranges of SO_2_. Multiple *in vivo* studies by our lab and others have shown concordant evidence in support of the Warburg effect in 4T1^2,38^.

Previous work has highlighted the peritumoral area (PA) as the site of intensive angiogenesis in human head and neck cancers^55^, and fibroblasts from the PA have been shown to promote phenotypic changes in tumor cells^57^. Assessing the PA of each tumor group in our study revealed that abnormal vascular features indeed extended well beyond the tumor boundary. Key features we observed included increased vessel diameter, comparable to what our group observed during spontaneous carcinogenesis in a hamster cheek pouch model^72^. One notable difference from our hamster study was the increased vessel fraction that we observed here, likely because the tumors in our study were angiogenic, yet too small to have developed avascular or necrotic areas^73^. Interestingly, vessels in the 4T1 PAs were even more abnormal than in the corresponding subject-matched tumors that they neighbored.

Abnormal vascular features in the PAs prompted further comparison of metabolism in the PA and tumor areas, since angiogenesis has been shown to recruit cancer associated fibroblasts (CAFs)^56^. It was recently hypothesized that tumors act symbiotically with such fibroblasts to compartmentalize metabolism and decrease competition for resources^11^. 4T1 tumors have been shown to have higher levels of Wnt7a than 4T07 tumors, which promoted *in vivo* fibroblast recruitment and activation^74^. A separate study showed that co-culturing 4T1 cells with CAFs enhanced the growth of 3D tumor clusters^75^. The RWE has been directly observed in both the primary tumor and lymph node metastases of other metastatic breast cancers^76^, and increased glycolytic markers in the tumor microenvironment predicted for poor patient survival in clinical breast cancer^77^. A pre-clinical study of MDA-MB-231 breast cancer demonstrated that simulating the RWE by administering aerobic glycolysis by-products caused increased tumor metastasis compared to control^78^. In the same study, *ex vivo* tissue analysis of tumor and adjacent stromal tissue showed a transcriptional shift toward mitochondrial metabolism in the tumor tissue. We observed increased metabolic compartmentalization consistent with the RWE in metastatic 4T1 tumors, since glucose uptake was increased in the PA and MMP was increased in the tumor.

We sought to test the hypothesis that the RWE in 4T1 tumors would allow them to maintain mitochondrial activity during acute hypoxia, since a glycolytic PA could provide fuel for OXPHOS while decreasing competition for oxygen. Surprisingly, MMP was not only maintained, but actually increased in 4T1 tumors during an hour of forced hypoxia without significant change in the steep metabolic gradients between the PA and the tumor (Supplementary Fig. S5). MMP decreased in all other groups over the same period. Importantly, monitoring changes in SO_2_ and glucose during the hypoxic perturbation gave crucial context for interpreting the changes in MMP. In groups that had increased glucose uptake and decreased MMP during hypoxia (N.T., 67NR, and 4T07) decreases in SO_2_ were not significant, suggesting diminished oxygen consumption. On the other hand, the increase in MMP in 4T1 correlated with a drastic and significant decrease in SO_2_, consistent with increased oxygen consumption. Correlation between increased MMP and respiration has been seen during hypoxia in cell studies^14^. If there was no indication of oxygen consumption in our study as reflected by SO_2_, a transient increase in MMP could have indicated reverse proton-pumping which has been seen in the progression toward hypoxia-induced necrosis in colorectal cancer cells^79^. This underscores the importance of measuring multiple metabolic endpoints, as well as concomitant changes in SO_2_, for appropriate interpretation of metabolic behavior.

A previous study showed that a loss of Bnip-3 protein expression in 4T1 cells enabled them to escape hypoxia-induced apoptosis^53^. Whereas cleaved caspase-3 (suggestive of apoptosis) increased after 6 hours of hypoxia in 67NR and 4T07 tumors, it decreased in 4T1 tumors during the same time period. During hypoxia, increased HIF-1α is known to upregulate expression of Bnip-3, toward mitochondrial autophagy^10^. Since Bnip-3 can impair respiration and cause a loss of MMP^80^, there may be a link between downregulated BNIP3 in 4T1 and maintenance of hypoxic mitochondrial function. A recent study found increased PGC-1α expression in 4T1 cells exposed to hypoxia, and saw that PGC-1α was responsible for increased mitochondrial biogenesis and metabolism^15^. Optical imaging of the redox ratio of endogenous FAD and NADH fluorescence also detected increased mitochondrial metabolism in 4T1 cells after exposure to hypoxia and increased glycolysis in the 67NR cells, consistent with our results shown in Fig. 6^81^.

Distinct mitochondrial responses to hypoxia were also recently seen in a panel of hepatocellular carcinomas and primary hepatocytes, where particularly aggressive cancer cells, but not primary cells, showed an increase in MMP and oxidative enzymes during hypoxia^14^. In that study, however, aggressive tumors also showed stark hypoxia-induced increase in glycolytic enzymes, which would be consistent with known HIF-1-dependent metabolic changes^10^. Other studies have shown that glutamine, and not glucose, is the major carbon source for OXPHOS-driven ATP production in cancer cells even during hypoxia^82^. Interestingly, an *in vitro* study showed that succinate dehydrogenase expression increased in 4T1 during hypoxia, but only when glutamine was available^2^. This finding, along with our observation that 4T1 tumors increased MMP yet decreased glucose uptake during hypoxia, may point to glutamine as an alternate fuel for mitochondrial metabolism during hypoxia *in vivo*, contrary to *in vitro* 4T1 behavior that was strongly glycolytic during hypoxia^2^.

Further study is required to determine the reason why metabolic compartmentalization affects only the 4T1 tumors, while all tumors had comparable vascular phenotypes. Because our study looks at tumors at an early time point (7–10 days), we are likely detecting a metabolic shift that precedes overt vascular changes in 4T1. The finding would not be surprising, since metabolic changes can be attributed to multiple factors which can be either independent from or related to neovascularization. Increased metabolism preceding the recruitment of new vessels in cancer would be consistent with published literature of diverse tumor types^83^, and pyruvate produced by glycolysis is known to be pro-angiogenic^84^. Further, studies have seen that dietary restriction can greatly reduce tumor vascularization^85^, supporting the role of metabolism in vessel growth. On the other hand, the changes may share a common upstream regulator; for example, expression of HIF-1α transcription factor can cause both metabolic and vascular changes^86^. It should be noted, though, that the glycolytic products lactate and pyruvate have been shown to be necessary for accumulation of HIF-1α^87^, and this phenomenon has interestingly been shown to affect oxidative but not aerobically glycolytic cells^88^. The precise relationship between cancer’s metabolic and vascular changes and the timing of each continues to be a subject of much interest, and we hope that our study contributes to the body of knowledge on this topic.

Similarly, while there were clear differences in metabolism that correlated with metastatic potential in this study, additional studies are needed to determine whether these findings can be generalized beyond the 4T1 family to other triple-negative breast cancers (TNBCs) or tumor types with varying metastatic potential. In contrast to the 4T1, an *in vitro* study of TNBC line MDA-MB-231 showed that the MDA-MB-231 cells maintained high levels of glycolysis through metabolic complementarity with oxidative CAFs^89^. A separate study showed that circulating tumor cells isolated from *in vivo* models of MDA-MB-231 and 4T1 had enhanced mitochondrial respiration, with no significant change in glycolysis, relative to the primary tumors^15^. The enhanced mitochondrial activity mediated through PGC-1α was required for metastatic dissemination. Recently, Neveu, *et al*., showed that *in vitro* MDA-MB-231 cells were glycolytic and insensitive to changes in oxygenation; in the same study, they showed that *in vivo* MDA-MB-231 tumors reduced glycolysis upon exposure to carbogen breathing^71^. Taken together, these studies indicate that consideration of not only the cell line, but also the experimental model and oxygen status, is crucial for metabolic phenotyping.

Regarding our technique, the following cautions should be considered. As discussed above, the TCA cycle in tumors can be fueled by a host of different carbon sources including glucose (following glycolysis)^90^, glutamine (following glutaminolysis)^90^, and fatty acids (following fatty acid oxidation)^91^. Use of any of these substrates can maintain mitochondrial membrane potential. Though mitochondrial membrane potential captures an important downstream component of mitochondrial metabolism, a limitation of the endpoint is that it cannot distinguish which carbon source is being used in the TCA cycle. Information on metabolic heterogeneity, spatial relationships, and vascular morphology obtained from optical imaging can be coupled with information on metabolic pathway intermediates obtained from metabolomics or magnetic resonance spectral imaging to provide a truly holistic view of a tumor’s metabolic preferences.

Next, the use of an athymic nude mouse model cannot fully replicate the tumor microenvironment seen in an immune competent host. For this preliminary study, athymic mice were chosen so that we could phenotype a more homogeneous tumor cell population without confounding immune factors, while maintaining the presence of peri-tumoral fibroblasts^56^. Though optical imaging represents an inherent trade-off between resolution and penetration depth, the window chamber has proven an invaluable platform for studying metabolism and angiogenesis *in vivo* in breast cancer^92–98^. Tumors grown in this model develop hypoxia^47^ and interact with the existing host vasculature^99^. Further, our characterization in this study showed that the peri-tumoral area was characterized by angiogenesis and hyper-metabolism, features which have been seen in *ex vivo* studies of patient tumors^55,77^. Since a recent study showed that 4T1 and 4T07 cells have different metabolic properties when grown in high or low density matrix^100^, further investigation into the exact make-up of the tumor microenvironment in our model will be critical before comparison to other models. In this single-timepoint study we were unable to definitively distinguish between pre-existing and neovascular vessels; in future studies, subtraction of vascular images taken before and after tumor inoculation will enable characterization of each vessel subset. Lastly, for this study it was necessary to image 2-NBDG and TMRE endpoints in separate cohorts of mice to validate our method. Future work will focus on the integration of endpoints for holistic imaging on a single tumor, as well as calibration of both fluorescence endpoints to an objective energy measurement such as ATP production to allow calculation of a standalone “energy budget”. We believe that *in vivo* imaging of glucose uptake, MMP, and vascular features simultaneously in the same animal will prove useful as a complementary new technique for the study of tumor metabolism.

## Materials and Methods

### Ethics Statement

All *in vivo* experiments were conducted according to a protocol approved by Duke University Institutional Animal Care and Use Committee (Protocol A114-15-04).

### Murine cancer cell lines

Three murine mammary carcinoma cell lines arising from the same spontaneous murine breast tumor were used in the study^53,54,101^. 67NR and 4T07 are non-metastatic; 67NR fails to leave the primary site, and 4T07 disseminates cells but cannot form metastatic nodules^101^. 4T1 are highly metastatic to lung, liver, bone, and brain^101^. The 4T1 family is typically considered a triple negative breast cancer model (ER-/PR-/HER-)^75,81^, although nuclear estrogen receptor alpha positivity has been observed in 67NR^102^. The 4T1 and 4T07 cells were acquired from the American Type Culture Collection, and the 67NR cells were generously provided by Dr. Fred Miller (Karmanos Cancer Institute, Detroit, MI) through Dr. Inna Serganova and Dr. Jason Koucher (Memorial Sloan Kettering Cancer Center, New York, NY). Cell lines were passaged every 2-3 days in RPMI-1640 medium (L-glutamine) with 10% fetal bovine serum (FBS) and 1% antibiotics (Pen Strep). For *in vivo* injection, cells were prepared in sterile RPMI-1640 containing no FBS nor antibiotics.

### Imaging probes

TMRE reports mitochondrial membrane potential by accumulating in proportion to MMP and causing an increase in fluorescence intensity^58^. TMRE was chosen for the study because its superior mitochondrial binding and rapid equilibration^59^ are beneficial for *in vivo* experiments. For *in vivo* administration, TMRE (Tetramethylrhodamine Ethyl Ester, Life Technologies/ThermoFisher) was diluted to a final concentration of 25 μM in sterile PBS. 2-NBDG (2-(*N*-(7-Nitrobenz-2-oxa-1,3-diazol-4-yl)Amino)-2-Deoxyglucose, Duke University Small Molecule Facility) was diluted to a final concentration of 6 mM. The total volume of each *in vivo* injection was 100 μL. The 2-NBDG dose was optimized in a prior publication^38^. The TMRE dose was chosen to keep the final concentration of TMRE well below 50 nM at the tissue level so that it operates in non-quenching mode and causes the least disturbance to electron transport^45^. For the current study, an injection of 100 μL of 25 µM TMRE gave a final tissue-level TMRE concentration range of 3.8-13.4 nM in normal tissue and tumors, calculated from tissue-mimicking phantoms (not shown).

### Dorsal skin flap window chamber model

Titanium window chambers were surgically implanted on the back of 8-12 week old female athymic nude mice (nu/nu, Duke DLAR Breeding Core, Durham, NC) using an established procedure^103^. We injected a 50 μL suspension (~1 × 10^5^ cells) of 4T1, 4T07, or 67NR cells into the dorsal skin fold and placed a glass coverslip (dia = 12 mm, No. 2) over the exposed tissue. Tumors were allowed to grow for 5–7 days before imaging. N.T. window chambers received no cell injection. All tumors used for imaging in this study had a tumor volume <150 mm^3^. All animals were housed in an on-site housing facility with *ad libitum* access to food and water and standard 12-hour light/dark cycles.

### Hyperspectral imaging of metabolic and vascular endpoints

For a 6-hour period prior to imaging, the animals were only provided with water. At the end of 6 hours of fasting, we initially recorded trans-illumination (vascular) images and corresponding background fluorescence images. In addition, a free space trans-illumination image using appropriate neutral density filters was recorded after every imaging session to account for daily variations in light intensity. The animals were administered a 100 μL tail-vein injection of 25 μM TMRE or 6 mM 2-NBDG in sterile PBS, and fluorescence images were captured for 75 minutes. We used a Zeiss Axioskop 2 microscope fitted with an LCTF, previously described in detail^50^, for all imaging. A 2.5 × objective lens (NA = 0.075) was used to yield a FOV of 9 mm with a resolution of 17.5 μm. Imaging excitation/emission wavelengths were 540 ± 20 nm/590 ± 5 nm for TMRE and 470 nm ± 20 nm/525 nm ± 5 nm for 2-NBDG.

An image acquisition time of 600–800 ms was used for both 2-NBDG and TMRE imaging. All fluorescent images were background fluorescence-subtracted and calibrated for integration time with a Rhodamine B standard solution prior to data analysis or visualization. During baseline (normoxia) measurements, the animals were allowed to breathe 21% oxygen. For the hypoxia group, the animals were subjected to breathing 10% oxygen for 15 minutes prior to imaging and through the end of imaging. Animals were anesthetized under inhaled isoflurane (1–1.5% v/v) in room air or hypoxic gas for the duration of imaging.

Separate cohorts of animals also received treatment with 50 µM CCCP. CCCP (carbonyl cyanide 3-chlorophenylhy-drazone, Sigma Aldrich) was diluted to a final concentration of 50 μM in DMSO. After animals were anesthetized with inhaled isoflurane (1–1.5% v/v) in room air, the window glass was removed and 0.1 mL of CCCP was topically applied to the tissue. The glass was immediately replaced, and imaging began 5 minutes later, following the imaging procedure used for all other groups.

### Multiphoton imaging of TMRE in a window chamber

One N.T. mouse was imaged with an Olympus FV1000 Multiphoton microscope, which we have previously described^65^. Hoechst 33342 (bisBenzimide H33342 trihydrochloride, Cell Signaling Technologies) was diluted to a final concentration of 2 mg/mL in sterile PBS. To visualize nuclei, the mouse received a 100 µL injection of Hoechst 33342 subcutaneously in the window chamber 15 minutes prior to injection of TMRE. TMRE was injected via tail vein following the standard dosing procedure. We used a two-photon excitation wavelength of 900 nm and collection wavelength ranges of 420–460 nm for Hoechst 33342 and 575–630 nm for TMRE.

### Calculation of vascular and metabolic parameters

Transmission images were collected in 10 nm increments from 520–620 nm and used to create an image cube (x, y, λ). A modified form of the Beer-Lambert law was fit to the trans-illumination image cube (x, y, λ) to obtain the concentration of the primary absorbers, oxy [HbO_2_] and deoxy-hemoglobin [dHb], at each pixel^47^. We then calculated total hemoglobin content, [THb] ([HbO_2_] + [dHb]), and SO_2_ ([HbO_2_]/[THb]) at each pixel. A binary mask was created from the presence or absence of [THb] in the transmission image cube, and used to segment the images into vascular and tissue space, respectively. Other vascular endpoints (length, diameter, tortuosity, area fraction) were quantified from the binary mask with in-house software that was extensively validated elsewhere^104^. The vessel diameter was calculated at each vessel midpoint, and all diameters within a region of interest were averaged to obtain a mean vessel diameter. The vessel fraction was defined as VF = (#vascular pixels/#total pixels) in the region of interest.

For tumor groups, the data shown corresponds only to pixels within the tumor region and excludes the surrounding tissue. Since increased scattering (due to cellularity^105^) and increased absorption (due to angiogenesis^106^) are well-known optical properties of tumors, we used regions of decreased light transmission in the 540 nm white light images to identify tumor tissue. Further, visual inspection and palpation of the tissue side of the window chamber aided clear identification of tumor regions. To avoid bias, hand traced masks were created for each tumor region by viewing the white light (transmission) images only, prior to viewing the corresponding metabolic endpoint images (2-NBDG, TMRE, or SO_2_). Further demonstration of the masking procedure is provided in Supplementary Fig. S1. Due to low excitation efficiency near the edge of the window chambers, tissue less than 1 mm from the chamber edge was masked using an automated process and excluded from analysis; this process was applied to all groups.

We chose to study TMRE uptake at 60 minutes (TMRE_60_) to correspond in time to the previously validated glucose uptake endpoint 2-NBDG_60_/R_D_ (2-NBDG uptake at 60 minutes corrected by a delivery factor^38,39^). We confirmed that TMRE signal was stable over the imaging period and responsive to known perturbations of MMP (Figs 1 and 2). Pixels in the tissue space of TMRE_60_ or 2-NBDG_60_/R_D_ images were used to create a pixel distribution curve (1-cumulative distribution) for each animal, and the individual pixel distribution curves were averaged to create the curves shown (mean ± SE). Area under the curve (AUC) was calculated for each mean pixel distribution curve.

TMRE_60_ and 2-NBDG_60_/R_D_ were additionally parsed by SO_2_. For each TMRE_60_ or 2-NBDG_60_/R_D_ image, every tissue pixel in the tumor area was assigned to an SO_2_ group according to the SO_2_ of the nearest vascular pixel. In a given image, there were as many as three SO_2_ bins: 0–20% SO_2_, 20–40% SO_2_, and 40–60% SO_2_. The distribution of TMRE_60_ or 2-NBDG_60_/R_D_ pixel intensities within each SO_2_ group was represented as a pixel distribution curve. Each final curve then represents the mean distribution (±SE) of TMRE_60_ or 2-NBDG_60_/R_D_ values in a given SO_2_ “bin”, for all mice containing image pixels in that SO_2_ bin.

### Statistical analysis

The cohort sizes were based on an expected average change in mitochondrial membrane potential between normal and tumor tissue based on previous literature (N.T. MMP ≈ −140 mV [3], Tumor MMP ≈ −200 to −220 mV [4, 5]). The study was powered to detect a small range of Cohen’s d effect sizes between d = 1.5 and d = 2. These effect sizes correspond to a standard deviation of 52 (for d = 1.5) to a standard deviation of 40 (for d = 2.0). Our data has a standard deviation around 50, which gives an effect size d ≈ 1.6 and minimum sample size n = 6 for each group, assuming normality and group means of 220 and 140, to attain 80% power at a 5% level of significance. Mean metabolic and vascular properties were compared with one-way ANOVA tests of log-transformed data. Tukey-Kramer post-hoc tests were used for all ANOVA. A two-sided t-test was used to compare normoxic and hypoxic conditions for a single endpoint. A paired t-test compared animal-matched tumor and PA vascular properties or animal-matched SO_2_ at baseline and post-hypoxia.

Whenever reasonable, we used the distribution of all pixels from each image to increase the number of data points for our analysis. For comparisons of pixel distribution curves, we used a repeated measures Kolmogorov–Smirnov test, which does not make an assumption of independence for pixels arising from the same mouse. Empirical p-values were calculated for the Kolmogorov-Smirnov statistic using blocked permutation (n = 1000 random permutations per test), prior to binning data for graphing. Error bars show standard error. P-values are indicated as * for p < 0.05. MATLAB (MathWorks, USA) Statistics Toolbox was used for all tests. One 67NR SO_2_ was excluded as a statistical outlier (determined by Grubbs test, α = 0.05) and one 4T1 hypoxia SO_2_ was excluded due to a recorded experimental error; no other data was thrown out.

### Data availability statement

The datasets generated during and/or analyzed during the current study are available from the corresponding author on reasonable request.

## Electronic supplementary material

Supplementary Figures and Methods
