# Supplementary material for: Metaboloptics: Visualization of the tumor functional landscape via metabolic and vascular imaging
Source: Sci Rep. 2018 Mar 8;8:4171. doi: 10.1038/s41598-018-22480-w (PMC5843602; doi:10.1038/s41598-018-22480-w)
Supplement: Supplementary file 1 — Supplementary Figures and Methods [file 41598_2018_22480_MOESM1_ESM.pdf]

Metaboloptics: Visualization of the tumor functional landscape via metabolic and vascular imaging

Authors

Amy F. Martinez[1]*, Samuel S. McCachren III[1], Marianne Lee[1], Helen A. Murphy[1], Caigang Zhu[1], Brian T. Crouch[1], Hannah L. Martin[1], Alaattin Erkanli[2], Narasimhan Rajaram[1], Kathleen A. Ashcraft[3], Andrew N. Fontanella[1], Mark W. Dewhirst[3], Nirmala Ramanujam[1]

Affiliations

[1] Department of Biomedical Engineering, Duke University, Durham, NC, USA

[2] Department of Biostatistics and Bioinformatics, Duke University Medical Center, Durham, NC, USA

[3] Duke University Medical Center, Durham, NC, USA

Contact Information

*Department of Biomedical Engineering

1427 FCIEMAS, 101 Science Dr.

Campus Box 90281

Durham, NC 27708-0281

Phone: 919-660-8473

Fax: 919-684-4488

Email: amyfmartinez@alumni.duke.edu

Supplementary Materials and Methods

Seahorse extracellular flux assay

A Seahorse Mito Stress Test (Agilent/ Seahorse Biosciences, Massachusetts, USA) was used to measure the metabolic properties of 67NR, 4T07, and 4T1 cells. Immediately prior to the experiment, all wells were replenished with Seahorse XF Base Medium supplemented to contain final concentrations of 12.5 mM glucose and 2 mM glutamine. The plate was then incubated in an oxygen-free environment for 1 hour. Oxygen consumption rate (OCR) and extracellular acidification rate (ECAR) were measured under baseline conditions and after perturbation with oligomycin and Carbonyl cyanide-*4*-(trifluoromethoxy)phenylhydrazone (FCCP). OCR and ECAR for each well were normalized by the cell confluence of the well. Results represent the average of 10 total wells from two assays that were performed on different days.

Supplementary Figures

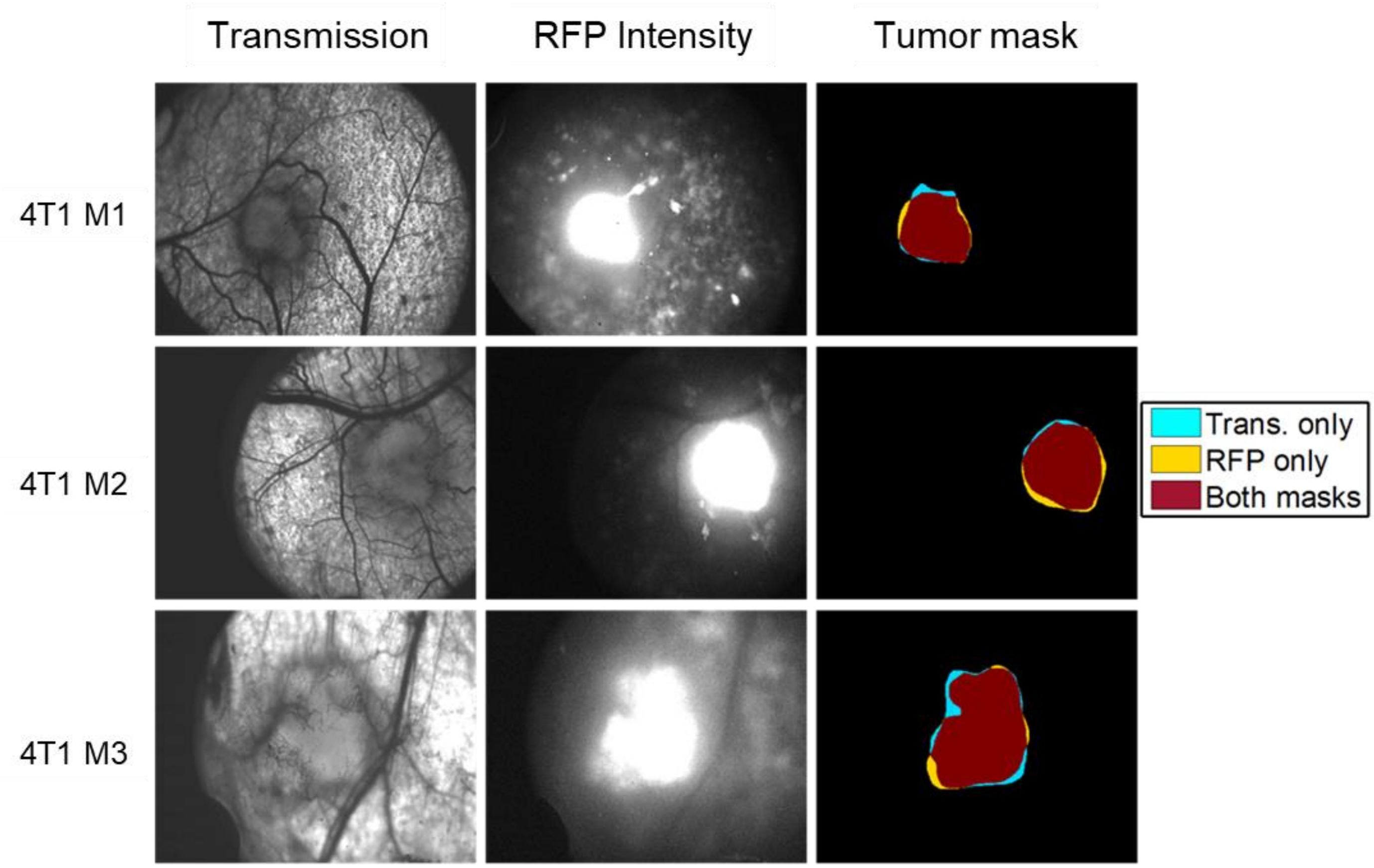

	Pixels in Trans. mask only	Pixels in RFP mask only	Pixels in both masks	% error
**M1**	5063	4010	69354	13.1%
**M2**	3020	8935	93470	12.8%
**M3**	14013	6467	152319	13.4%

Supplementary Figure S1. Comparison of tumor masks created from transmission and RFP images. Transmission and RFP fluorescence intensity images were obtained for three mice bearing RFP-tagged 4T1 tumors. Hand traced masks were created from each image, and the overlay of masks created from transmission and RFP images is shown in the right column. Red indicates the area of agreement for both masks, and yellow or blue regions were traced in only the RFP or transmission images, respectively. The disagreement between masks (percent error) was ~13% for each tumor.

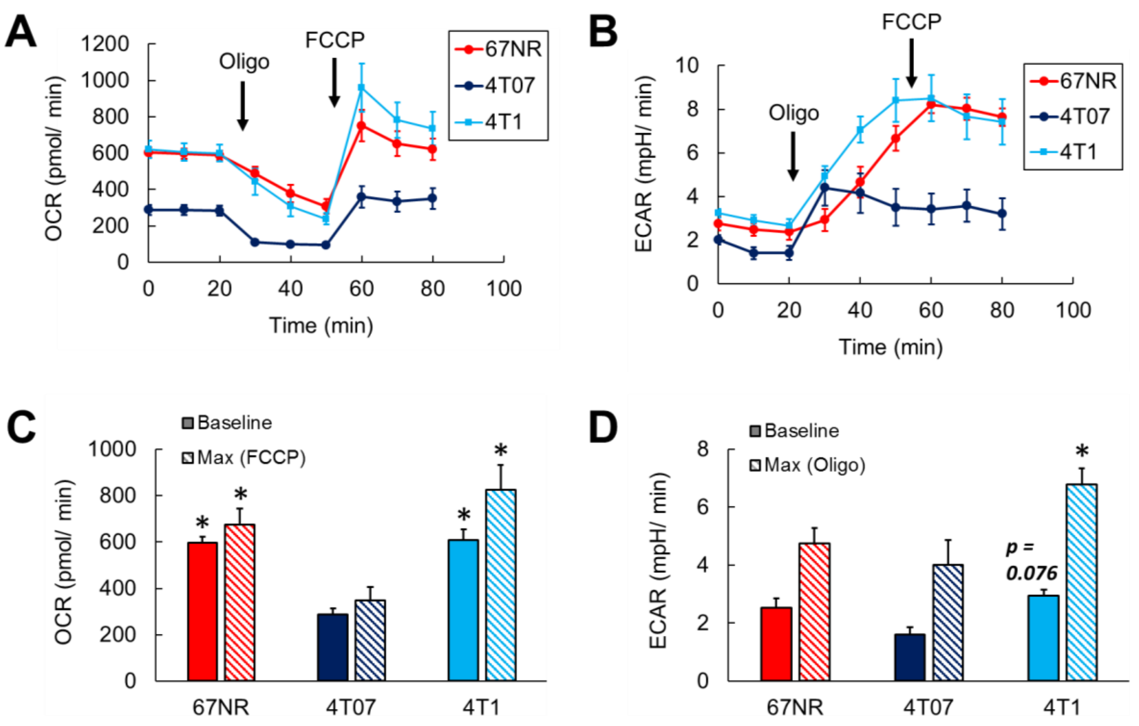

Supplementary Figure S2: *In vitro* characterization of 67NR, 4T07, and 4T1 cell lines. A, Oxygen consumption rate (OCR) and **B,** extracellular acidification rate (ECAR) measured at baseline and after addition of oligomycin (Oligo) and FCCP. **C**, Mean oxygen consumption rate at baseline (solid) and after addition of FCCP (striped). **D,** Mean extracellular acidification rate at baseline (solid) and after addition of oligomycin (striped). Each mean is an average of three time points: OCR or ECAR baseline (0, 10, 20 min), OCR max (60, 70, 80 mins), ECAR max (30, 40, 50 mins). n = 10 wells/ group. Error bars = SE. * is $p<0.05$ vs. 4T07; all other comparisons NS.

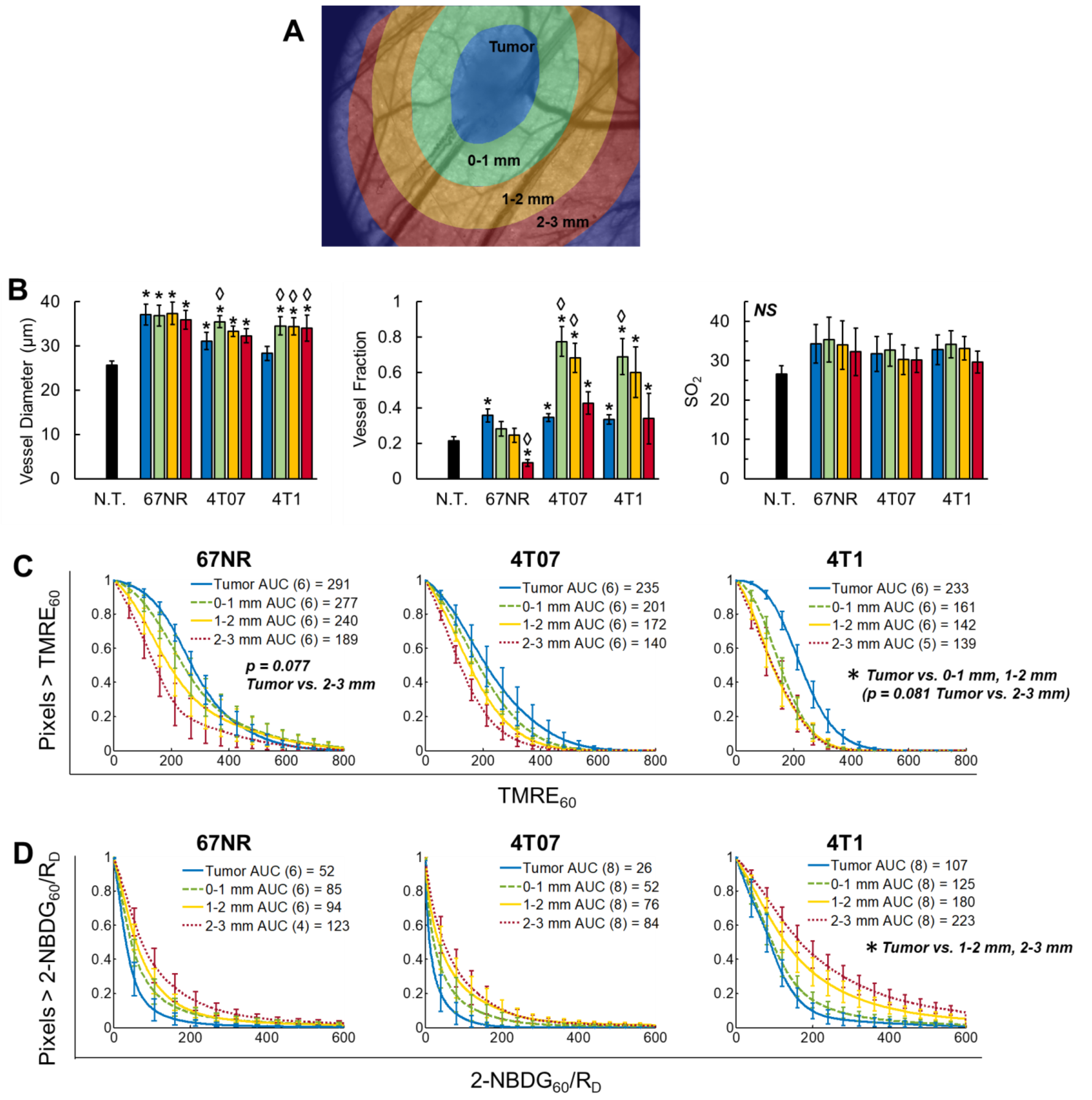

Supplementary Figure S3: Distance from the tumor border affects the metabolic phenotype of the peritumoral area. Metabolic and vascular endpoints were quantified for the tumor and three zones in the peritumoral area (PA) of all tumor types. **A,** Transmission images were segmented into tumor and three PA regions. The three regions contained pixels 0-1 mm, 1-2 mm, or 2-3 mm from the tumor border. **B,** Comparison of mean vascular features in non-tumor (N.T.) tissue and tumor and all PA regions of all tumor types. SO_2 = vascular oxygenation. **C,** Distribution of $TMRE_{60}$ pixels in the tumor and PA regions of all tumor types. **D,** Distribution of 2-$NBDG_{60}/R_D$ pixels in the tumor and PA of all tumor types. AUC = area under curve. n = 11-14 (B) or as shown in legend (C,D). * is p<0.05 vs. N.T. (B). ◊ is p<0.05 tumor vs. PA region for same tumor line (B). * is p<0.05 tumor vs. PA for same tumor line (C,D).

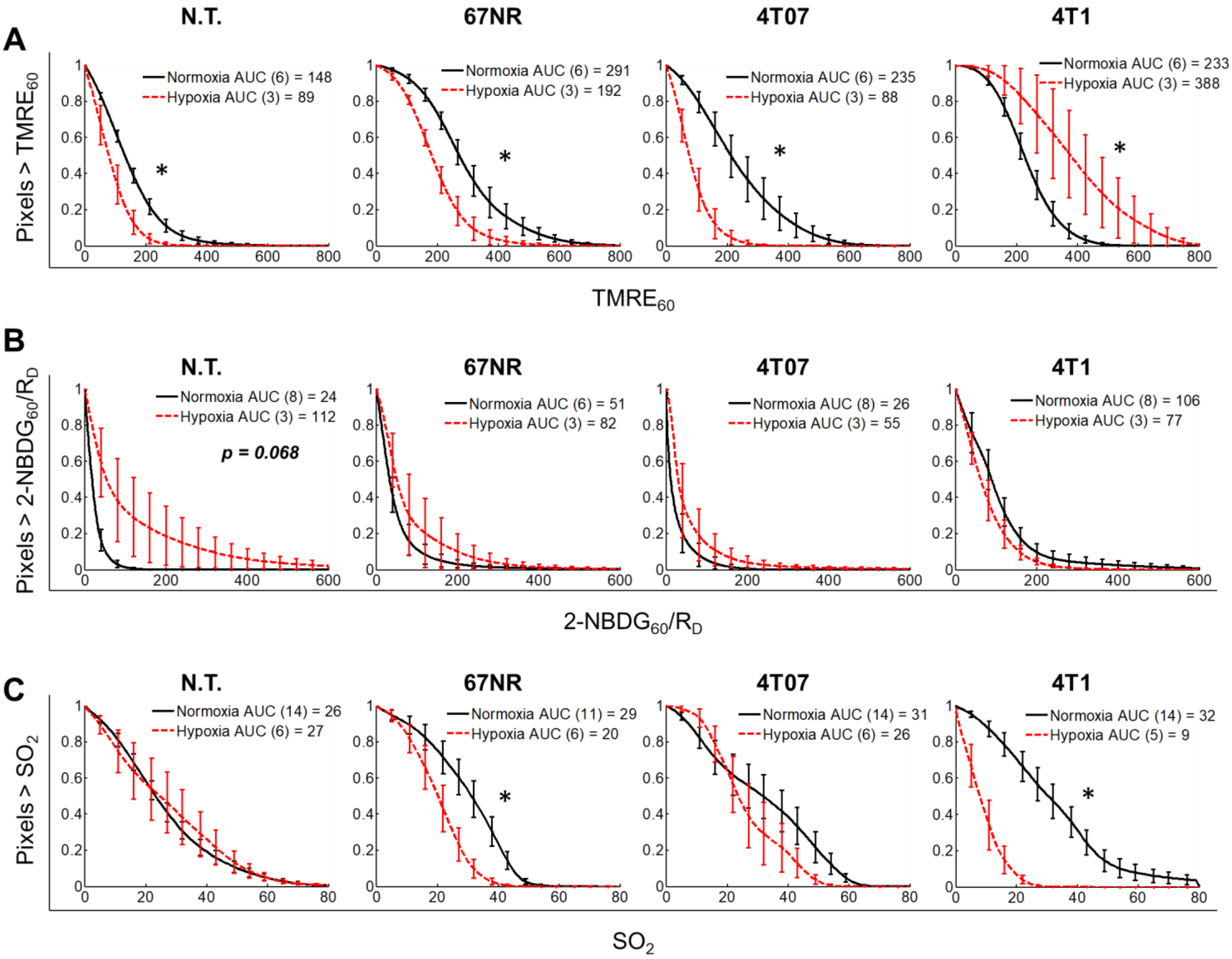

Supplementary Figure S4: Changes in metabolic endpoints during forced hypoxia. Mean distributions of all $TMRE_{60}$ (**A**), 2-$NBDG_{60}/R_D$ (**B**), and SO_2 (**C**) pixels for each group during normoxia and hypoxia. Group numbers shown in legend. N.T. = Non-tumor. AUC = area under curve. Error bars = SE. * for $p<0.05$

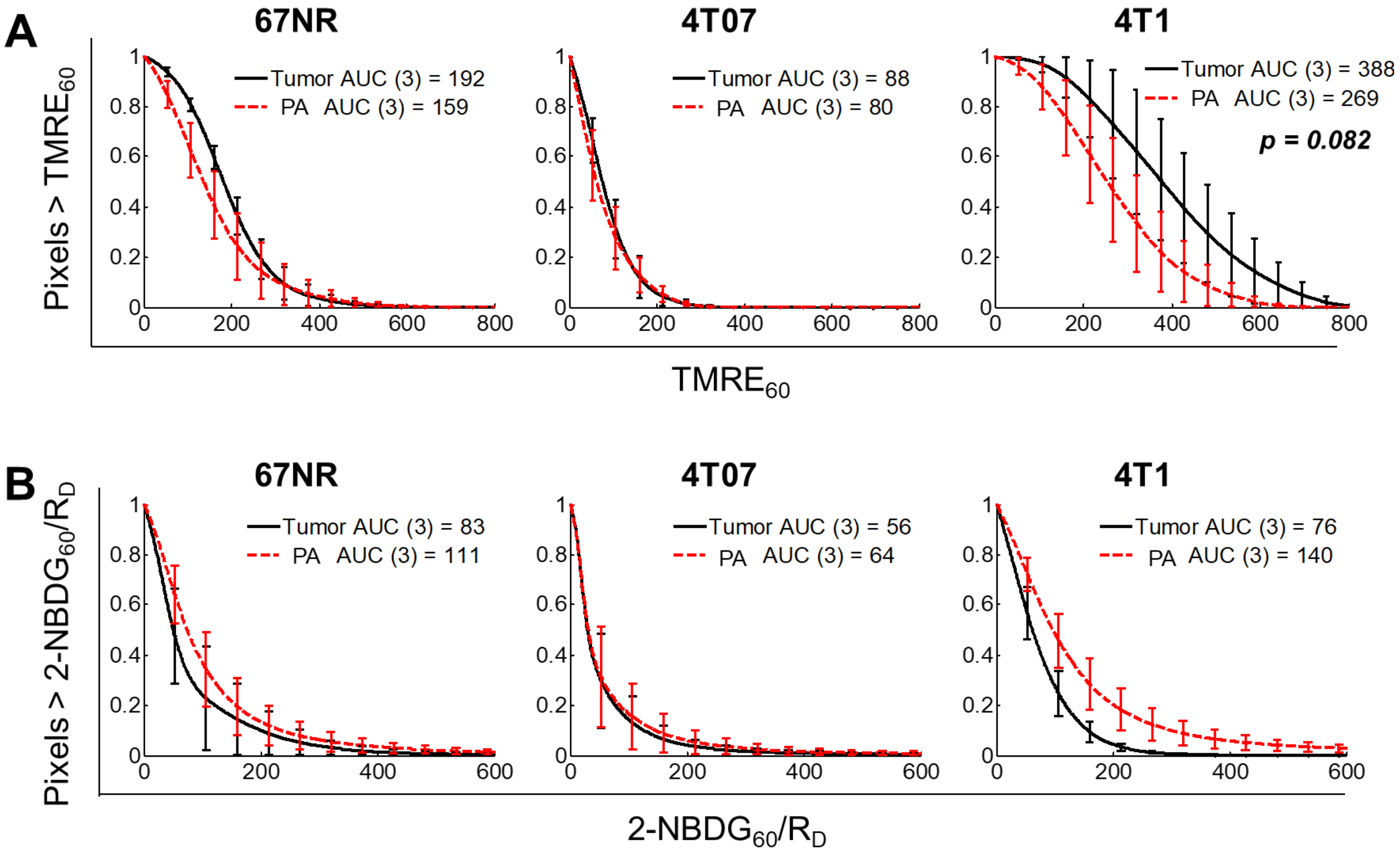

Supplementary Figure S5: Metabolic compartmentalization is seen in 4T1 during forced hypoxia (10% inspired O_2). A, TMRE$_{60}$ in the tumor and PA of all tumor types during hypoxia. **D,** 2-NBDG$_{60}$/R$_D$ in the tumor and PA of all tumor types during hypoxia. AUC = area under curve. n = 3.
